# Plant‐based foods as meat and fat substitutes

**DOI:** 10.1002/fsn3.3421

**Published:** 2023-05-11

**Authors:** Claire D. Munialo, Frank Vriesekoop

**Affiliations:** ^1^ Food, Land and Agribusiness Management Harper Adams University Newport UK

**Keywords:** alternative proteins, environmental footprint, fat substitutes, functional characteristics, meat substitutes, plant‐based foods, sustainability

## Abstract

Animal proteins have in the past been used in food production due to their functional properties that range from gelation and emulsification to foaming ability and stability. However, animal husbandry has been shown to be a major contributor to global warming and climate change. Consequently, there has been a drive toward the use of alternative proteins, for example, proteins from plant sources which are perceived to be cheaper, healthier, and sustainable. The use of *trans* and saturated fatty acids in the food industry has been associated with various health issues that include an increased risk of metabolic disorders. This has resulted in an increased search for fat substitutes that are healthier and sustainable. To contribute toward a reduction in the consumption of meats from animal sources and the consumption of *trans* and saturated fatty acids, the formulation of plant‐based meat and fat analogs/substitutes has been carried out. However, there has been a lower acceptance of these meat or fat substitutes which was attributed to their sensorial and textural properties that fail to mimic or resemble real fat or meat. Therefore, this review aims to discuss the advances that have been made when it comes to plant‐based meat and fat substitutes. Additionally, consumer perception and acceptance of these products will be reviewed as well as future markets will be discussed and the opportunities and challenges that exist in the formulation of these products will be explored.

## Graphical abstract

Plant‐based protein alternatives are considered to be more sustainable than animal proteins. Plant‐based proteins are suggested to have lower nutritional quality than animal proteins. Plant‐based meat or fat substitutes need to mimic their animal‐based counterparts in terms of texture and sensorial attributes for consumer acceptance.

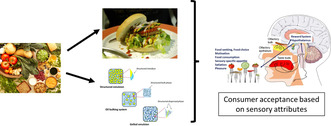

## 1 INTRODUCTION

The global population continues to increase with recent models from the United Nations showing that the world's population recently reached 8 billion people (Le Page, 2022). There is a further estimation that the world population will reach an estimated 9.8 billion people by 2050 (Desa, 2019), which will continue to add pressure to the food industry to ensure the supply of enough food including protein to feed the growing population. This heightens the search and need for reliable and sustainable protein sources.

Proteins from both plant and animal sources have been used in food processing. Animal proteins have been used in food production for decades because of their techno‐functional properties. In this review, techno‐functional properties are defined as the ability to form and/or stabilize networks (films and gels), foams, emulsions, and solutions in addition to water‐ and oil‐holding capacities (Shokri et al., 2022). However, animal husbandry has been associated with an increase in the production of greenhouse gases (GHG) which have been shown to contribute to global warming and climate change. For instance, a recent GHG lifecycle assessment showed the current livestock production systems to contribute to the global anthropogenic GHG emissions by approximately 18%. This did account for 65% of anthropogenic nitrous oxide and a total of 37% of anthropogenic methane (Patra, 2014). The dairy cattle sector was shown to contribute to 4% of total anthropogenic GHG emissions, whereas beef production was reported to contribute to the most GHG emissions. Some authors suggest a global average of 50 kg of GHG to be released per ~100 g of animal protein produced (González et al., 2011). Additionally, a diet rich in meat has been related to an increased risk of human health issues (such as noncommunicable and metabolic disorders like cardiovascular diseases, hypertension, and obesity among others), which is mainly attributed to the high content of cholesterol and saturated fatty acids (Boukid, 2021). As such, there has been an increase in research to find affordable alternative protein sources that have a lesser impact on climate, reduced risk toward noncommunicable diet‐related diseases, and lower mortality (Springmann et al., 2018).

Proteins from plant sources such as pea (Munialo et al., 2015; Munialo, Martin, et al., 2014; Munialo, van der Linden, & de Jongh, 2014), soy (Geerts et al., 2018; Lee et al., 2016; Li et al., 2020; Preece et al., 2017), and lupin (Berghout et al., 2014; Lo et al., 2021) among others have been extracted and characterized in terms of their techno‐functional properties (such as gelation, emulsification, and foaming) as potential replacements for proteins from animal sources. However, some authors (Berrazaga et al., 2019; Pinckaers et al., 2021) have reported plant‐based proteins to have less of an anabolic effect compared to animal proteins. This was mainly attributed to their lower digestibility and lower content of essential amino acids (EAAs), particularly leucine, in addition to their deficiency in other EAAs, for instance sulfur‐containing amino acids or lysine. Furthermore, the techno‐functional properties that play an important role in giving food its appealing texture and sensory attributes for animal proteins are considered superior to proteins from plant sources (Kim et al., 2022). Thus, plant‐based proteins are often considered inferior when compared to their animal counterparts.

Despite the superior techno‐functionality of proteins from animal sources, their usage has been associated with climate changes as aforementioned and hence there has been a drive to look for alternative sustainable alternative sources. However, to be able to reduce the use of proteins from animal sources, there is a need to identify proteins that can mimic the functionality of animal proteins. This has been of great significance when it comes to the use of plant‐based proteins as structural ingredients which can be assembled, for example, in plant‐based meats or used as fat substitutes and thus would be used as a versatile alternative to animal proteins. These attributes are affected by a myriad of factors such as the quality of the protein in addition to the functional properties of the alternative proteins.

Excessive consumption of a diet that is characterized by fat, in particular, *trans* and saturated fats, has been linked to adverse effects on health. Additionally, the consumption of *trans* and saturated fats has been correlated to an increased risk of chronic diseases such as cancers, cardiovascular diseases, obesity, and type 2 diabetes, among others (Yashini et al., 2021). However, the presence of fat in food is desirable as it improves the characteristics of the food product. Fat contributes toward sensorial attributes and textural properties of food products and this impacts palatability and mouthfeel and consequently consumer perception and acceptability (Rios et al., 2014).

There has been a drive to look for fat replacers or fat substitutes to replace fat in food products as a strategy for reducing the consumption of *trans* and saturated fats. Fat substitutes replace fat molecules in the food with components that have comparable properties. As such, both animal and plant‐based protein–fat replacers have been explored and researched for their potential for use in the food industry. However, the drive toward sustainability, cost, and health among others has made it even more vital for researchers and the food industry to design plant‐based fat substitutes.

Therefore, this article aims to review the use of plant‐based foods as meat and fat substitutes. The protein quality of plant‐based foods, their functional properties, the assembly and processing into the meat and fat substitutes, as well as challenges and opportunities that exist in the use of plant‐based proteins in the formulation of these substitutes will be discussed.

## 2 AN OVERVIEW OF PLANT‐BASED FOODS

There is a myriad of plant‐based foods that have been formulated and are available in the market with various trademark names. The formulation of these foods, however, does depend on various factors such as the quality of the protein as well as the techno‐functional characteristics that impact their application and this will be reviewed next.

### 2.1 The protein quality of plant‐based foods

Protein plays various roles in the human body such as (i) regulation of gene expression, (ii) modulation of the immune system in addition to (iii) comprising the major structural elements of all cells and (iv) being involved in the formation of the major constituents of muscles (Lieberman, 1999). Thus, proteins are deemed to be a vital nutritional constituent. However, one principal thing to always consider is the fact that the overall nutritional value of a protein‐rich food is not represented by protein alone as in most cases protein analysis does omit the assessment of the accompanying macro‐ and micronutrient composition (McAuliffe et al., 2023). One other limitation of focusing on proteins only is the fact that this does omit some complexities such as the presence of antinutritional factors (e.g., phytates and oxalates) which are often present in plants and have the potential of interfering with protein digestibility (Munialo & Andrei, 2023).

There have been several schools of thought regarding the nutritional quality of plant‐based foods with some authors proposing the distribution of amino acid profile of proteins from plant sources to be less optimal than in foods made from animal‐based protein. The content of EAAs such as lysine is present in grains in proportions that are much lower than optimal for human needs and methionine and cysteine which are sulfur‐containing amino acids being slightly lower in legumes than what would be considered optimal for human needs (Mariotti & Gardner, 2019). Other authors have argued that plant‐based proteins have lower nutritional values which was attributed to an imbalance in the composition of amino acids (Berrazaga et al., 2019; Pinckaers et al., 2021), in addition to a slow or reduced digestibility which has been attributed to their molecular structures (Day et al., 2022). However, most scholars have come to the consensus that when appropriate dietary requirements are considered, most people can source all the amino acids requirements from plant‐based foods which would make a significant contribution to their health and livelihood as they enter the adult phase of their lives, and as such, proteins from plant sources can still offer a good source of protein and contribute to a balanced diet for humans (Mariotti & Gardner, 2019; Sá et al., 2020; Schweiggert‐Weisz et al., 2020).

Table 1 shows the nutritional values of EAAs from major foods that are consumed across the globe. Some of the plant‐based sources of food contain a considerable amount of these amino acids with pea and nuts being comparable to most animal protein sources. Soybeans are listed to contain a lower level of several EAAs compared to proteins from animal sources. Additionally, tofu seems to have a lower content of most of the amino acids in comparison to most of the animal proteins, pea, wheat, and nuts which could be attributed to the processing that soy undergoes to formulate tofu and this could result in some losses of the amino acids. Thus, the processing steps need to be considered when looking for or selecting proteins that can be used as replacements for animal proteins during the processing of meat or fat substitutes. Moreover, there is a need to ensure that the amino acid profiles of the proteins are (i) bioavailable, (ii) digestible, and (iii) well balanced in order to ensure that they meet the dietary requirements of any given individual. Several strategies can be used to modify proteins from plant sources which in turn would contribute to the enhancement of the amino acid profile that includes increased bioavailability and digestibility. The adjustment of the pH, enzymatic treatments, fermentation, as well as heat treatment could be some of the ways to enhance the bioavailability of the amino acids. These modification strategies and their impact on the amino acid profiles of proteins have been discussed in detail elsewhere (Nikbakht Nasrabadi et al., 2021). For instance, the application of high pressures and temperatures during extrusion has been reported to have the potential of destroying antinutrients and improving the digestibility of proteins from plant sources as the availability of their amino acids is increased (Chen et al., 2018). Additionally, there could be the potential of using a mixture of plant‐based proteins which are different in their amino acid profiles and their interactions in different matrices could enhance the content of amino acids. However, there is still a need to understand how these function at a molecular level and translate to a product development level. Moreover, an exploration of the potential for the use of modification strategies for the enhancement of amino acid content in proteins from plant sources needs to be carried out. The other issue that needs to be considered is the allergenicity of some of these plant‐based proteins which have the potential of causing adverse immune‐mediated reactions which are commonly known as food allergies (Turnbull et al., 2015). Thus, this is something that needs to be addressed and looked into in detail before these proteins can effectively be used as replacers for animal proteins.

**TABLE 1:** Nutritional value of EAAs (g/100 g of raw product) from major foods consumed across the globe (Haytowitz et al., 2019).

	Wheat	Tofu	Soybeans	Peas	Nuts	Pork	Eggs	Cheese	Beef
Isoleucine	0.23	0.32	0.81	0.98	0.75	1.03	0.67	1.21	0.92
Histidine	0.16	0.19	0.45	0.59	0.54	0.89	0.31	0.55	0.71
Leucine	0.45	0.50	1.36	1.68	1.47	1.77	1.09	1.94	1.70
Lysine	0.22	0.43	1.12	1.77	0.57	1.95	0.91	1.03	1.86
Methionine	0.14	0.08	0.22	0.20	0.16	0.58	0.38	0.55	0.55
Phenylalanine	0.30	0.32	0.87	1.15	1.13	0.88	0.68	1.07	0.80
Threonine	0.20	0.27	0.72	0.81	0.60	0.96	0.56	1.04	0.91
Tryptophan	0.07	0.10	0.24	0.16	0.21	0.24	0.18	0.55	0.21
Valine	0.28	0.33	0.83	1.04	0.86	1.12	0.86	1.40	0.99

### 2.2 Techno‐functional properties of plant‐based foods

The use of protein ingredients to formulate plant‐based products is dependent on their techno‐functional properties that include their solubility in water, foaming ability and stability, gelling properties, water‐ and oil‐holding capacities, and emulsifying potential (Kyriakopoulou et al., 2021).

The molecular structure as well as the ability to interact and form complexes by food macromolecules (such as lipids, proteins, and polysaccharides) makes them inherently functional. The molecular structures of proteins have significant roles in determining the functionality in food, and consequently, they can be used as targets in altering protein functionality (Aryee et al., 2018). Intrinsic factors (such as the amino acid composition, the conformation, surface functional groups, protein structure, net and surface electric charge, in addition to hydrophobicity or hydrophilicity) and environmental/extrinsic factors (such as the pH of the medium, the ionic strength, salts and solvents, shear stress, temperature, and pressure) all determine the stability, the shelf life, as well as the techno‐functional properties of foods that contain functional proteins (Kyriakopoulou et al., 2019). The functional properties of proteins can also be modulated by how proteins are extracted or processed. Either wet or dry fractionation has been used in protein extraction and the protein yield, as well as the functionality of the protein extract have been reported, and this has been discussed elsewhere (Pelgrom et al., 2013; Schutyser et al., 2015).

The globular nature of most proteins contributes to their functionality, in particular, the solubility, which has been attributed to the amphiphilic nature of these molecules (Barac et al., 2015). Proteins contain both inwardly bound amino acids that are hydrophobic (apolar) and outwardly side chain of amino acid residues that are hydrophilic (polar). This arrangement makes it possible for dipole–dipole interactions with solvents to occur due to the side chains of amino acids twisting and unfolding, which places the polar groups at the surface of the protein. As a result, networks are formed which can either form gels or develop films, absorb fat, form foams, contribute to emulsification, hold water, as well as dissolve under various pH conditions (Aryee et al., 2018). Other factors such as the relative number of α helices, random coils, as well as the ratio of α helices and β sheets that are present in the secondary structures of corn meal and soybean protein have also been reported to positively correlate with the solubility of the protein, whereas the percentage of the β‐sheet structures was shown to negatively correlate with this same ability (Bai et al., 2015).

Some studies have assessed the techno‐functional properties of proteins from plant sources, such as chickpeas, kidney beans, and soybean (Byanju et al., 2020), pea and mung beans (Xiong et al., 2018), lentils (Liu et al., 2018), cowpea (Teko et al., 2022), and amaranth and quinoa (Ruiz, 2016) among others. However, it is noteworthy mentioning that utilization of proteins from plant sources is oftentimes limited due to their solubility which tends to be extremely low at neutral pH, except proteins from cowpea, canola, pea, and soybean (Sá et al., 2022). The limited solubility can be the result of processing, such as lyophilization, which results in structural and conformational changes in the proteins (Munialo et al., 2022).

The foaming ability and stability of some plant‐based proteins such as chickpea, lupine, soybean, pea, and rapeseed have also been evaluated (Tontul et al., 2018). These proteins from plant sources have been shown to exhibit excellent foaming ability and stability, which were comparable to egg protein, predominantly because of their low molecular weight, high solubility, and high surface hydrophobicity, in addition to the net charge (Sun‐Waterhouse et al., 2014).

Some proteins from plant sources, such as bell pepper (Li et al., 2018), peas (Barac et al., 2015), and chickpeas (Karaca et al., 2011), have been shown to display emulsifying properties as they formed stable emulsions with small oil droplet sizes that had high emulsion activity index at pH 10 (Tontul et al., 2018). Relatively high emulsifying ability and emulsion stability against creaming during storage of soybean and rapeseed have also been reported (Chen et al., 2011).

A number of studies have evaluated the water‐ and oil‐holding capacities of plant‐based proteins. Bell peppers were shown to be ideal in food products that require a relatively high ability to hold water (Li et al., 2018), while the ability of peanut protein isolates to hold oil was considerably higher than commercial soybean protein isolates (He et al., 2014). These findings widen the window of applications of proteins from plant sources and reduce the overdependency on soybean proteins which have been associated with several issues that will be addressed later on in this review.

Even though the gelation properties of plant‐based proteins have been evaluated in a number of studies, on the one hand, some of these proteins, for example, rapeseed concentrates, isolates, and flours, have been reported to have poor gel formation properties (Tan et al., 2011). On the other hand, soybean protein isolates have been shown to possess a higher ability to form gels and as a result have been used as gelling agents in several semisolid food products mainly designed for meat analogs (Bessada et al., 2019).

In conclusion, it is worth noting that, in terms of techno‐functionality, there is limited research that has reported the combination of the solubility, emulsifying, foaming, water‐ and oil‐holding capacities, and gelling properties of, for example, a single protein from plant sources, as most research usually focuses on one or two functional properties of any given protein at a time. However, proteins from plant sources have the potential of being used by the food industry in formulations for meat analogs, beverages, protein supplements, and snacks, among other products (Kyriakopoulou et al., 2019). As such, the exploration of plant‐based proteins in terms of their techno‐functional properties should be considered with the aim of developing a database of technological alternatives for food formulation. Thus, there is still room for further research that includes the evaluation of the processing technologies that are required for extraction and modulation of the techno‐functionalities of proteins from plant sources. This will provide insight into different protein structure–function interactions, for instance, in terms of their hydration and solubility, the gelation, and the formation of texture, all of which govern the final structure of final product such as meat analogs.

## 3 PLANT‐BASED MEAT SUBSTITUTES

The reduction of the consumption of meat can be established through various ways such as (i) the use of plant‐based alternatives to partially replace meat‐based products or via the application of a “less but better” principle where less quantity but more quality environmentally and/or animal‐friendly meat is consumed, (ii) the reduction in the portion sizes of meat that are consumed, (iii) the replacement of meat with another protein source that could range from foods from animal sources (such as eggs or cheese) to the consumption of alternatives that can be derived from plants (such as legumes mushrooms, or tofu) or the use of other alternative protein sources which currently have a minimal (i.e., insects and seaweed) or nonexistence market share (i.e., laboratory cultured meat), (iv) the complete elimination of meat from the diets, and last but not least, (v) the consumption of plant‐based meat alternatives (PBMAs; Andreani et al., 2023; Dagevos, 2016).

The market for plant‐based meat analogs has continued to boom, going from “niche” to more “mainstream,” with more than 6000 new product launches worldwide since 2015 (Boukid, 2021). Plant‐based meat analogs which are also referred to as “faux meat, meat substitutes, or mock meats” are plant‐based products that are designed in such a way that they mimic the appearance, flavor, and fibrous texture of animal meat (Andreani et al., 2023). Several nonanimal protein sources such as cereals, vegetables, legumes, microalgae, and fungi have been used in the substitution of animal proteins to produce a wide spectrum of products that are meat‐free such as sausages, burger patties, and nuggets (Saget et al., 2021).

The major components of fibrous meat analogs are made up of 20%–50% plant proteins, 0%–5% vegetable lipids, and 2%–30% polysaccharides in addition to other ingredients that enhance a meat‐like appearance. A summary of protein ingredients that are used for meat analog applications is provided in Table 2. Based on the examples that are provided, there is an opportunity for other proteins of plant origin to be explored in terms of the possibility of them being used in the production of meat analogs given the myriad of alternative proteins that have been investigated and extracted for use in the food industry. This is particularly the case for soy‐based products given the competition that exists in the production of soy for both animal and human consumption, hence if other plant proteins can be explored for their use in the production of meat substitutes, the pressure on the production of sustainable soy will be minimized. Additionally, there are allergenicity issues that are related to soy proteins which provide a window of opportunity to search for soy replacers with less or no allergenic potential.

**TABLE 2:** A summary of protein ingredients that are used for meat analog applications (Kyriakopoulou et al., 2021).

Composition (% w/w)	Protein ingredient	Functionality	The application in meat analogs		
			Structuring process	Role	Products
~70% Protein	Soy concentrate	Good texturization properties	Extrusion, shear cell	Binder, protein source, and texture	Burger patties, minced meat, muscle‐type products, sausages
~90% Protein	Soy isolate (alkaline/acid precipitation)	Good emulsification, gelling, and solubility	Extrusion, freeze structuring shear cell, spinning	The base for fat substitutes, binder, emulsifier, protein source, texture	Burger patties, minced meat, sausages
~90% Protein, denatured due to heat treatment	Soy isolate (additional heat treatment/toasted isolate)	Decreased solubility, increased water‐holding capacity, good gelling	Extrusion, shear cell	Protein source, texture, binder, a base for fat substitutes	Burger patties, minced meat, sausages
~43%–56% Protein, ~0.5%–9% fat, ~3%–7% crude fiber, >30% total carbohydrate	Soy flour/meal (defatted)	Water‐binding capacity and fat retention, native protein	Extrusion	Binder, texture	Burger patties, minced meat, muscle‐type products, sausages
>45% Protein, ~30% fat	Soy milk (spray‐dried powder)	High solubility, good emulsification properties	Freeze structuring	Emulsifier, texture	Tofu and yuba production
~85% Protein	Pea isolates	Water and fat binding, emulsification, and firm texture after thermal processing	Extrusion, shear cell, spinning	Binder, emulsifier, texture	Burger patties, minced meat, muscle‐type products, sausages
75%–80% Protein, 15%–17% carbohydrates, 5%–8% fat	Wheat Gluten isolate	Binding, low solubility, the formation of dough, cross‐linking capacity via S–S bridges	Extrusion, shear cell	Adhesion, texture	Burger patties, muscle‐type products

[Corrections added on 26 May 2023, after the first online publication; Table 2 was corrected to include wheat gluten isolate].

## 4 THE PRODUCTION OF PLANT‐BASED MEAT ALTERNATIVES

Plant‐based meat alternatives seem to fit better in the “niche” category, however, in general, the use of alternative proteins appears to have transformed from being a niche product to a mainstream phenomenon (Szenderák et al., 2022). In the past, the available PBMAs included tofu and tempeh from soy and seitan from gluten. The 21st century meat alternatives used the crosslinking capacity of soy proteins under certain conditions of soy proteins in the fabrication of these products. To date, soy remains to be the main raw material that is used to produce meat alternatives (Zhang et al., 2021). The soy supremacy indubitably does depend on the raw material availability in addition to the techno‐functional attributes of its proteins, which include soy's ability to absorb water and oil, its emulsification potential and gelling propensity, as well as its solubility, which are all vital aspects when it comes to defining of the quality of the finished product (Nishinari et al., 2014). However, there has been a shift in the use of other raw materials instead of soy which was attributed to issues concerning allergies as aforementioned. Furthermore, adverse long‐term health consequences have been associated with the consumption of soy‐based infant formulas during developmentally sensitive windows (Ma et al., 2022). Other issues influencing the use of soy include GMOs, unfavorable climate for the cultivation of soy, and the valorization and/or the preservation of biodiversity (Andreani et al., 2023). Consequently, recent work has explored the use of proteins that are sourced from different raw materials, such as peas, fava beans, rapeseeds, and hemp, which has been done without or in combination with soybean as discussed in detail elsewhere (Grossmann & Weiss, 2021; Ma et al., 2022). However, these alternatives have been shown to not be able to satisfy the texture of meat especially among western consumers (Andreani et al., 2023).

Alternatives from plants have been made from protein extract where processes such as wet extraction have been used to produce either protein isolates or concentrates. The protein isolates or concentrates have then been fabricated into meat analogs using a myriad of processes such as electrospinning, wet spinning, extrusion, and flow‐induced structuring using a shear cell or a Couette cell and this has been discussed in detail elsewhere (Andreani et al., 2023). There are also novel technologies such as three‐dimensional (3D) printing which have been used to digitally model the formulation of food and texture to mimic the meat matrix that is found in beef (Godoi et al., 2016). Start‐ups such as Novameat, Redefine Meat, and Aleph Farms have been launched to produce 3D printed plant‐based meat, “which has textural resemblances like real muscle tissue and taste like meat” (Singh et al., 2021). Even though 3D printed faux steaks are not yet fully available on the market, the main challenge of 3D meat printers remains to be the scalability process, the production cost, the maintenance services, the complexity of the spatial structure, in addition to regulatory frameworks which include but is not limited to allergens, adulteration, labeling, and culinary creativity (Godoi et al., 2016).

## 5 THE DEVELOPMENT OF PLANT‐BASED FAT SUBSTITUTES

In the past, partially hydrogenated oils (PHOs) were extensively used in food processing. However, in recent years, there has been a shift toward the removal of these oils from food products. This removal of these fats is an attempt to eliminate industrially produced *trans* fats from the food supply, given that PHOs presently represent the most significant dietary source of artificial *trans* fatty acids (Wang et al., 2016). Thus, food companies have been faced with the challenge of finding viable substitutes for PHOs that mimic their functionality in food processing without impacting organoleptic properties and consumer acceptance. This is a significant technological challenge particularly in certain food systems which depend on the unique functional properties of such fats (such as the plasticity or laminating shortenings which are used in puff pastries and the specific melting profiles in confectionary fats; Wang et al., 2016). Ongoing research has focused on the substitutes that can be used to replace PHOs without impacting on the texture and organoleptic properties of the final product and some of the approaches that have been used will be discussed next.

### 5.1 Food polymer oleogels

Oleogels (also referred to as organogels or structured oils) are solid‐like lipid‐based materials which are composed of a large amount of oil that has been structured by oleogelators to form a three‐dimensional thermoreversible gel network (Shi et al., 2014; Stortz et al., 2012). Most structurants that can form networks fall under the category of either low‐molecular‐weight organogelators (LMOG) or polymers (Adams, 2022). Polymers have been used in many food applications, given that many are food grade as well as inexpensive compared to the highly purified LMOGs (Stortz et al., 2012). The continuous liquid phase of edible oleogels is composed of edible oils which are mostly structured by oleogelators and possess physicochemical properties that are similar to solid fats (Li et al., 2022). The distinct structures and physical properties of edible oleogels make them potential replacers of solid fats (which are relatively high in saturated and *trans* fatty acids). Saturated and *trans* fatty acids have been associated with an increased risk of several diseases as aforementioned. These include cardiovascular diseases (CVD), breast and colonic cancer, as well as other metabolic disorders such as diabetes and obesity. *Trans* and saturated fats have also been thought to increase allergies, the shortening of the pregnancy period, risks of preeclampsia, disorders of the nervous system in addition to impaired vision in infants (Dhaka et al., 2011). Most of the saturated and *trans* fatty acids are found in many commercial products (such as bakery, confectionery, and meat products). However, as more people are becoming aware of the impact of these fats on health, it has now become important for the food industry to search for alternatives and/or substitutes for these fats in food product formulations. Some authors have suggested that replacing 5% of fat with polyunsaturated fats, such as vegetable oils, has the potential of reducing the risk of CVD in humans by up to 22%–37% (Roche, 2005). It is, however, worth noting that *trans* and saturated fats are mainly added to foods because they provide technological and functional characteristics such as crispness, flavor, longer shelf life, and satiety which can be challenging to replace (Silva et al., 2023). Several methods that can be used to modify lipids (such as interesterification, fractionation, and blending) have been explored to substitute saturated and *trans* fats in foods. Albeit these methods have failed to satisfactorily reduce or eliminate the levels of *trans* and saturated fat without altering the characteristics of the product (Menaa et al., 2013). This can partly be related to the inherent differences that exist between fats and oils, some of which are due to certain characteristics that exist both in the structure as well as their functional properties.

Palm and palm kernel have extensively and successfully been used in foods to partially replace hydrogenated fats. However, there is an increasing concern about the use of these fats, as they have been shown to have a high saturated fatty acids content and as well as their use has been highly criticized and has been under a lot of scrutiny due to sustainability issues (Silva et al., 2023). Starch and other polysaccharides (natural gums) have also been studied and used to replace shortenings entirely or partially in confectionery products (Banaś & Harasym, 2021; Colla et al., 2018). However, the replacement of saturated fats with refined carbohydrates does not give the same characteristics to the product (Silva et al., 2023), and this can affect the final quality attributes, and consequently, consumer acceptance of the final product. Furthermore, the use of carbohydrates does not meet the current nutritional demands, given that diets that are composed of mono‐ and disaccharides with low‐fat and highly refined content have been associated with different health problems, such as dyslipidemia which is a component of the metabolic syndrome (Tanti et al., 2016). Thus, the search for lipid sources that have the technical functionality and can meet the new nutritional needs continues to be a necessary and unceasing challenge for the food industry (Silva et al., 2023).

Several structuring agents have been researched for edible oil structuring. These include monoglycerides (Da Pieve et al., 2010), lecithin (Si et al., 2016), phytosterols (Bot et al., 2009), and vegetable waxes (Da Pieve et al., 2010) among others. The combination of these gelators has also been researched by several authors (da Silva & Danthine, 2022; Godoi et al., 2019). These diverse molecules form basic supramolecular assemblies that fall into one of the following predefined oleogel structuration groups: (i) low‐molecular‐weight compounds that have the potential of forming self‐assembled structures; (ii) crystalline particles; (iii) polymeric or polymers strands of self‐assembled structures; and (iv) miscellaneous structures that include emulsion droplets and colloidal particles (Patel & Dewettinck, 2015). The high structuration that is promoted by different oleogel routes and the potential to mimic the high saturated behavior of fat has been performed on several food products, such as bakery products (Giacomozzi et al., 2018; Pang et al., 2022), confectionary (Kim et al., 2022; Patel, Rajarethinem, et al., 2014), ice cream (Roy et al., 2022; Zulim Botega et al., 2013), meat products (Barbut et al., 2016; Ferro et al., 2021), and margarine and spreads (Hwang & Winkler‐Moser, 2020; Öǧütcü & Yılmaz, 2014) among others. The application of these structuring agents in food systems has been discussed in detail elsewhere (Silva et al., 2023). However, it is worth noting that even though several studies have been done, only a few have been able to achieve all the requirements for a successful replacement of fat which includes 100% replacement with good technological properties, acceptable in terms of their sensory attributes, and economically viable. Hence, more research is still needed to find successful replacements that are organoleptically acceptable by the final consumer and yet still reasonable in terms of cost.

Several combinations of oleogelator and oil have been tested based on their textural properties and sensory attributes on different food products, and the results showed the potential of oleogels being used as a solid fat replacer. The used oleogelators and their concentrations have been reported to play a major role in the final application which is based on their interactions with other ingredients in the food matrix, such as water and sugar (Silva et al., 2023). Monoglycerides and vegetable waxes as oleogelators are viable fat substitutes, especially in margarines and spreads as well as in baked products where some successful full replacements of the physical, chemical, and sensory properties were found (Colla et al., 2018). Studied oleogels in meat products are also advanced with various polymers and proteins showing some favorable results (Gómez‐Estaca et al., 2019). Although for the confectionary and dairy products some promising results (such as ethyl cellulose and monoglycerides in chocolates, waxes in ice cream and chocolate paste, and monoglycerides in fillings) have been published. However, there is a need for more studies to be carried out that will confirm the findings especially with regard to shelf life, heat stability, and sensory acceptance if the goal is to achieve 100% replacement (Silva et al., 2023). Additionally, more studies on scaling up some of the technologies that have been used in the fabrication and testing of the techno‐functional properties of oleogels need to be carried out to pave way for their application in food product manufacture.

### 5.2 Protein–polysaccharides oleogels

Proteins are mainly used in the formulation of various food products because of their techno‐functional properties such as their excellent solubility in water under a wide range of pH and nutritional value. Proteins also can form well‐structured aqueous gels under defined physicochemical conditions which depend on the secondary structure as well as their amino acid sequence (Li et al., 2022). Gelation is the result of an out balance of attractive and repulsive interactions that often lead to protein aggregation. This process is governed by several factors such as heating and cooling rate, the ionic concentration, and pH variation as closer to the pI results in coarser networks, whereas away from the pI, finer protein networks tend to be formed (Munialo et al., 2015, 2018; Munialo, Martin, et al., 2014; Munialo, van der Linden, & de Jongh, 2014). Fibrillar or globular proteins are the two kinds of proteins that are mainly needed in the formation of oleogels. The gelation of globular proteins such as soy proteins is mainly driven by heat denaturation which is influenced by intermolecular interaction energy (Li et al., 2022). Heating of globular proteins results in the energetic motion of peptide chains which leads to the interaction between amino acids which bind to other proteins resulting in the formation of a three‐dimensional network. Animal proteins such as whey (Feichtinger & Scholten, 2020; Pinto et al., 2021) and egg proteins (Jaberi et al., 2020) have been used in the preparation of protein‐based oleogels. Soy protein (Naji‐Tabasi et al., 2020; Tavernier et al., 2017) and canola protein isolate (Li et al., 2022) are some examples of proteins of plant origin that have been used in the preparation of oleogels. However, there still exist challenges in the use of plant proteins to fabricate oleogels. Generally, the functionality of plant‐based proteins, such as emulsifying potential, solubility, gelling or foaming ability, as well as water‐holding capacity, is often considered inferior compared to proteins from animal sources as aforementioned. These differences arise from their distinct native environment (Feichtinger et al., 2022). Consequently, a direct substitution or replacement of animal proteins by plant proteins is usually challenging. A typical example of a challenge that arises when it comes to the substitution of animal proteins with plant proteins is their solubility. Proteins from plants are typically storage proteins and they generally have larger and more compact structures, in addition to being more hydrophobic and consequently also less soluble (Feichtinger et al., 2022).

Feichtinger and Scholten (2020) and Feichtinger et al. (2022) attempted to create plant‐based protein oleogels. The plant‐based protein oleogels showed comparable properties as animal‐based proteins in similarly efficient ways. The complexation between proteins (such as soy protein) and polysaccharides (such as kappa‐carrageenan) has also been used in the creation of oleogels (Manzoor et al., 2022). The complexation of protein and polysaccharide occurs through noncovalent bonding, and this leads to the formation of nanoparticles that have well‐defined dimensions and good dispersity (Qiu et al., 2018). The complexes that are formed between the protein and the polysaccharides once incorporated in the gel matrix can trap oil, which does promote the interaction between oil droplets, and this provides higher structural integrity (Qiu et al., 2018). Some authors have shown the interfacial rigidity and the network strength to be the key determinants for the polymer‐based emulsions to resist coalescence or even the separation of oil during dehydration (Patel, Cludts et al., 2014; Romoscanu & Mezzenga, 2006). As such, polymer complexation provides an ideal way to improve emulsion stability. Additionally, the interaction between proteins and polysaccharides in solution and at the oil–water interface can allow for unique hydration, structure, and surface properties (Qiu et al., 2015), which can further be applied in several domains, such as emulsion gels, hydrogels, and oleogels (Qiu et al., 2018). Even though the structural integrity of the oleogels has been elucidated, there remains a gap in understanding the digestibility of these gels as well as the stability of the oils against lipid oxidation when used in various processes that would require high heat given that oleogels are thermoreversible.

## 6 CONSUMER PERCEPTION AND ACCEPTANCE OF PLANT‐BASED MEAT AND FAT SUBSTITUTES

The market potential for meat substitutes continues to grow. In the year 2020, plant‐based meat sales at a retail level were shown to reach USD 4.2 billion globally which was a 24% growth in comparison to 2019 (GFI, 2021). Despite the changes in the market share of meat alternatives, the acceptance of novel and unfamiliar foods by the consumer is still a challenge to market stakeholders with the consumer acceptance of meat substitutes being shown to still be low or uncertain in several countries which could be related to food neophobia. Food neophobia is a typical response that comes into play when new technologies are used in the production of food or when food is deemed to defy the food and/or gastronomic cultures of consumers (Safdar et al., 2022).

Consumer perceptions about plant‐based meats have been shown to vary greatly across the world, which was attributed to cultural, behavioral, taste, as well as food habits differences (Safdar et al., 2022), while also considering the notion of “value for money” (Dean et al., 2023). In general, there is a paucity of research on consumer acceptance and preferences of meat alternatives, with some authors suggesting that “the portfolio of foods on the market that could realistically be regarded as a plant‐based equivalent to beef to be narrow” (Goldstein et al., 2017). However, it is important to note that the main factor that determines the success of any food product including plant‐based meat and fat substitutes is consumer acceptance.

Recent surveys revealed that only a small minority of consumers frequently purchase meat alternatives (Siegrist & Hartmann, 2019), whereas the majority do not consider meat substitutes (Lemken et al., 2019). Positive reactions toward meat alternatives were reported to come mainly from consumers who regularly ate meat alternatives. Regular consumers of meat alternatives rated them as better than meat, while those who moderately consumed meat alternatives gave balanced ratings, even though they were more positive about meat, whereas individuals who did not consume meat alternatives rated meat as being much better than meat alternatives (Michel et al., 2021). Hoek et al. (2011) have shown that consumers who favor the consumption of meat prefer meat alternatives that are similar to meat; contrastingly, the more people are in favor of the consumption of meat alternatives, the less they want these alternatives to resemble meat.

The other factor that will drive the growth of the plant‐based meat industry is the perception of these meats to be a threat by some people and communities who mainly depend on animal husbandry either for their livelihoods or commercial viability (Safdar et al., 2022), even though the rise of the plant‐based industry may not pose a threat to the sustainability of the animal meat sector (Van Loo et al., 2020).

## 7 PREDICTION OF CHANGES IN DEMAND AND SUPPLY OF PLANT‐BASED MEATS AND FAT SUBSTITUTES BY 2050

There are predictions that the world population will grow even further to more than 9 billion by the year 2050 (Roberts, 2011). With the expansion and growth of the world population, there will continue to be an increase in the demand and search for nutritious food and in particular protein‐rich foods to feed the growing population. The demand for meat and meat products will inevitably continue to rise as the world's population grows. However, to reduce the burden of meat production from a dietary, environmental, and ethical point of view, switching to plant‐based meats could be a possible alternative, while the use of plant‐based fat replacers could be viewed as a better option for the prevention of cardiometabolic disorders and this could increase their demand.

The growth of the plant‐based meats market is predicted to accelerate in the coming years, and this emerging market seems to be well‐positioned for potential development and innovation (Safdar et al., 2022). However, it is worth noting that the rise or fall of the plant‐based meat and fat substitutes is subject to the price of the final product. One of the keys to the plant‐based meat industry has been suggested to depend on the ability of plant‐based meat industry to price its products to be more competitive to conventional meat (Katare et al., 2023). The capability of the plant‐based meat industry to scale up production is all dependent on a higher consumer acceptability of these meats which is dependent on price equality with animal meat (Safdar et al., 2022).

In conclusion, it does seem to be quite unlikely that people would entirely switch from animal meat to plant‐based meat. However, it may be that the primary purpose of plant‐based meats will be to contribute toward expanding protein demands rather than to completely replace animal meat products. There is also potential use of plant‐based meat to help feed people in poor countries as well as in disaster‐prone regions (such as earthquake zones and flood‐prone areas) as a way of fulfilling their protein needs, or in places where the food supply and preservation are not possible (Safdar et al., 2022). However, it is worth noting that the capability of PBMAs to satisfy protein demand in poorer or developing countries is strictly dependent on affordability.

## 8 OPPORTUNITIES, CHALLENGES, AND LIMITATIONS IN THE PRODUCTION AND CONSUMPTION OF PLANT‐BASED MEATS AND FAT SUBSTITUTES

Although it has been a long time since the launch of plant‐based meat substitutes, research has shown a lack of reduction in meat consumption and the shift toward the consumption of plant‐based meats is way too small and slow in comparison to reducing the intake of animal or the replacement of animal meat with plant‐based meat substitutes (Safdar et al., 2022). Even though plant‐based meat substitutes are considered a healthier alternative to animal meat, the uptake and consumption of these alternatives are still discouraged by some governmental organizations and people worldwide (Van Loo et al., 2020). This is mainly attributed to the negative perceptions about technologies that are used in the fabrication of these meats in addition to the use of artificial preservatives/additives as part of the structural ingredients in PBMAs. Some individuals also perceive PBMAs to be unnatural, and some of the resistance is focused on its production that involves a high degree of processing (Santo et al., 2020). Price is also one of the important factors that influence consumers buying intentions. The retail price of plant‐based meat alternative products has been and will continue to be a critical issue for their success and acceptability as aforementioned. The premiums of plant‐based meats are reported to be higher when comparing overall prices to conventional meat on a per‐pound basis. It has been suggested that on average, plant‐based meat is 2 times as expensive as beef, more than four times as expensive as chicken, and more than 3 times as expensive as pork per pound (GFI, 2020). The price difference can act as an impediment to many consumers' willingness to purchase plant‐based meat substitutes (Rombach et al., 2022).

Even though previous research on the nutritional quality and the production of plant‐based meat alternative products has shown that they contain natural plant‐based ingredients and are made using safe technologies, the question, however, remains as to whether plant‐based meat substitutes contain artificial flavors and ingredients or are they healthier and more natural than animal meat? In food processing, it is common that when one ingredient is being removed, there is a need to replace this with an alternative that has similar techno‐functional properties as the one that is being replaced. Most of the time when proteins from animals are removed and replaced with those of plant origin, given the fact that plant‐based proteins tend to have a lower solubility and nutritional value than those of animal origin, other additives that have various functional roles such as binding the ingredients together are always added during product formulation (Munialo, 2023). There is also the issue of taste where plant‐based proteins tend to have some odd flavor notes (Ismail et al., 2020), which results in the use of additives (with some additives being added to mask these off flavors and tastes). In general, the safety of food additives is something that has often been debated with some authors proposing the possibility of the accumulative effect of these additives over time and this could be worrisome. Additionally, the processing steps involved in the formulation of these substitutes can result in things like denaturation of the proteins which can alter the structure of the same and result in a reduction in the nutritional value (Abraha et al., 2018) of the end product where the protein of the “raw product” even though high could be very different from the final product which also would undergo some form of cooking. This could mean that in the end, the product that the consumers are being offered, even though does seem to be more environmentally friendly, yet could result in other nutritional deficiencies and negative impact on human health in the long run. Additionally, most of plant‐based meat and fat alternatives have been classified as ultraprocessed foods (UPFs) as they are produced industrially from processed plant‐based ingredients (Ohlau et al., 2022). The consumption of UPFs has been associated with an increased risk of obesity and other diet‐related noncommunicable diseases (such as cardiovascular diseases, cancers, and diabetes; Monteiro et al., 2018), and as such, healthy nutrition is substantially jeopardized.

It is worth noting that encouraging people to replace animal meat products completely or partially in their diet with plant‐based meat is still challenging due to a general lack of cooking knowledge and skills. Additionally, meat is still viewed and believed by many people to be an important and necessary part of a balanced and healthy diet which is mainly related to enjoyment, taste, and habit (Santo et al., 2020). Thus, one would then wonder if increasing awareness and educating the public is vital as this would then involve the modification of the dietary habits of people, and consequently, there will be more willingness to consume and purchase plant‐based meat and fat substitutes (Munialo, 2023). In general, the success of the meat and fat substitutes market is determined by how well these products are received by consumers globally. Thus, individual preferences concerning meat alternatives must be understood to reduce the demand for animal meat and promote the shift toward the consumption of PBMAs, given that transition of protein from higher consumption of meat to its reduction or substitution with plant‐based meats is highly dependent on consumer behavior (Safdar et al., 2022).

## 9 OUTLOOK AND CONCLUSION

It is becoming increasingly clear that the use of animal proteins in food production continues to add pressure to global warming and climate change. Therefore, research has been carried out on the exploration of alternative proteins that have also been used in the formulation of plant‐based meat and fat substitutes. These meat and fat substitutes tend to be perceived as more sustainable and healthier even though the texture and other organoleptic properties of these substitutes remains to be a challenge that needs to be improved to widen their application in food production. The issue of food waste also still needs to be addressed to ensure that the new products that are being formulated help to ensure an adequate supply of nutritious foods to the growing world population and this contributes to the UN's sustainable development goals.

## AUTHOR CONTRIBUTIONS

**Claire D. Munialo:** Conceptualization (equal); project administration (equal); validation (equal); writing – original draft (equal); writing – review and editing (equal). **Frank Vrieskoop:** Formal analysis (equal); writing – original draft (equal); writing – review and editing (equal).

## CONFLICT OF INTEREST STATEMENT

None.

## ETHICS STATEMENT

Ethics approval was not required for this research.

## Data Availability

Research data are not shared.
